# Molecular and Cellular Mechanisms of Action of Tumour Suppressor GAS5 LncRNA

**DOI:** 10.3390/genes6030484

**Published:** 2015-07-07

**Authors:** Mark R. Pickard, Gwyn T. Williams

**Affiliations:** School of Life Sciences, Huxley Building, Keele University, Keele ST5 5BG, UK; E-Mail: g.t.williams@keele.ac.uk

**Keywords:** GAS5, lncRNA, cancer, tumour suppressor, apoptosis, cell proliferation

## Abstract

It is increasingly recognised that lncRNAs play essential regulatory roles in fundamental biological processes and, consequently, that their dysregulation may contribute to major human diseases, including cancer. Better understanding of lncRNA biology may therefore offer new insights into pathogenetic mechanisms and thereby offer novel opportunities for diagnosis and therapy. Of particular interest in this regard is GAS5 lncRNA, which is down-regulated in multiple cancers, with expression levels related to both clinico-pathological characteristics and patient prognosis. Functional studies have further shown that GAS5 lncRNA both inhibits the proliferation and promotes the apoptosis of multiple cell types, and that together these cellular mechanisms of action are likely to form the basis of its tumour suppressor action. At the same time, advances have been made in our understanding of the molecular mechanisms of GAS5 lncRNA action in recent years, including riborepression of certain steroid hormone receptors and sequestration of miR-21, impacting key regulatory pathways of cell survival. Overall this accumulating knowledge has the potential to improve both the diagnosis and treatment of cancer, and ultimately patient outcome.

## 1. Introduction

The long non-coding RNAs (lncRNAs) are a major component of the human transcriptome, but many remain to be functionally annotated [1,2]. Nevertheless, regulatory roles in fundamental cellular processes have emerged for a number of these molecules in recent years, and it is increasingly recognised that major pathologies, such as cancer, are characterized by dysregulated expression of lncRNAs [1,2,3,4]. Indeed, it is now widely acknowledged that lncRNAs are likely to be of crucial importance in the pathogenesis of cancer; therefore, increased understanding of lncRNA biology may lead to novel and better approaches for the diagnosis and treatment of this important disease.

To illustrate these points, this review focusses on the relatively well characterized lncRNA, GAS5, and cancer. This lncRNA is a transcript of the *growth arrest-specific 5* (*GAS5*) gene, a non-protein coding gene, which was first isolated in 1988 in a search for novel tumour suppressors by subtractive cDNA cloning of genes which are preferentially expressed in growth-arrested cells [5]. As will be discussed here, more recent work has shown that GAS5 lncRNA is dysregulated in multiple cancers and confirm a tumour suppressor role for this molecule. Recent advances in our understanding of the cellular and molecular mechanisms of GAS5 lncRNA action will then be considered, before discussing how this knowledge may be harnessed clinically in the future for the improved diagnosis and treatment of cancer.

## 2. Gene Structure

**Figure 1:**
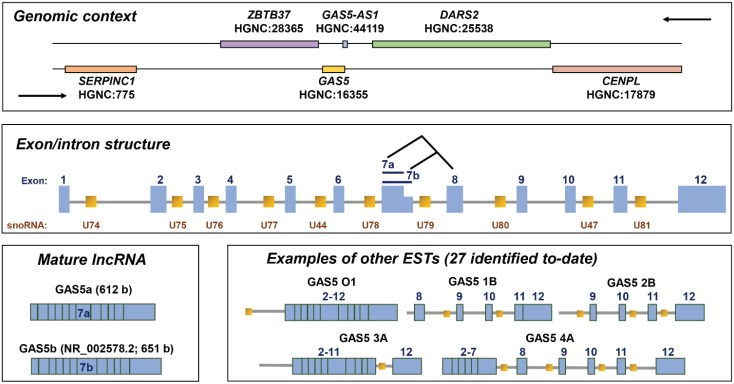
Genomic context, gene structure and selected products of the human *GAS5* gene. For genomic context, HUGO Gene Nomenclature Committee (HGNC) approved gene symbols are used, with the corresponding HGNC identity number given below each symbol. Exon/intron structure is based on published data [6]; note the presence of two alternative 5′-splice donor sites in exon 7. The two possible resulting mature lncRNAs are termed GAS5b (contains exon 7b and corresponds to the GAS5 reference sequence, NR_002578.2) and GAS5a (contains exon 7a); the latter is predicted to be 39 bases shorter than GAS5a (based on [6] and GenBank: AF141346.1). The additional *GAS5* expressed sequence tags (ESTs) shown here have all been reported to induce growth arrest in lymphoid cell lines [10].

*GAS5* is localized at 1q25.1 and encodes small nucleolar RNAs (snoRNAs), microRNAs (miRNAs) and PIWI-interacting RNAs (piRNAs), in addition to lncRNA [6,7,8]. The gene comprises 12 exons, which contain only a short open reading frame and are not thought to encode a functional protein; rather these exons are spliced to yield two possible mature lncRNAs, termed GAS5a and GAS5b, due to the presence of alternative 5'-splice donor sites in exon 7 (Figure 1) [6]. *GAS5* additionally encodes within its introns ten (human) or nine (mouse) box C/D snoRNAs, which participate in the 2'-O-methylation of rRNA; the U44 snoRNA serves as a guide to modify 18S rRNA, whereas the remaining snoRNAs all direct the modification of 28S rRNA [6]. Moreover, at least three of these snoRNAs—U44, U74 and U78—may give rise to miRNAs [7]. A 5'-terminal oligopyrimidine (5'-TOP) tract is present in exon 1 of *GAS5* [6]; this motif is commonly found in genes that encode proteins that function in ribosome biogenesis and translation. While the 5'-TOP sequence usually serves to control the translation of such proteins, in the case of *GAS5*, it serves to control transcript levels (see Section 4).

The processing of the *GAS5* is poorly understood but is likely to be complex, insofar as a multitude (29 at the latest count) of transcripts have been described [9]. Many of these contain retained introns and may exert isoform-specific functional effects [10,11]. Nevertheless, in our experience, mature lncRNA appears to be the predominant transcript in most cell lines, and particularly the GAS5b variant (Figure 1). Notably there is partial overlap of 40 or so of the 3' terminal nucleotides of GAS5 with another non-protein coding gene, *GAS5-antisense-1* (*GAS5-AS1*), which is encoded on the opposite strand and arranged tail-to-tail with *GAS5* (Figure 1). *GAS5-AS1* is largely uncharacterized and the impact of its transcription on *GAS5* expression is unknown.

## 3. Protein-Coding or Non-Protein Coding?

Recent studies have reported that certain lncRNAs give rise to small polypeptides or micropeptides [12,13]. Indeed, in the case of GAS5 lncRNA, this has been recognized for several years [6]. Thus, human *GAS5* may encode a 50 amino acid polypeptide whereas, for mouse and rat *GAS5*, 23 and 13 amino acid polypeptides, respectively, have been predicted [6,14,15]. With the exception of the 5'-TOP sequence and snoRNA-encoding intronic sequence, *GAS5* sequence has generally been thought to be poorly conserved between humans and rodents and even between rats and mice [6,14,15], albeit it is now recognized that discrete, functionally important portions of lncRNA sequence show greater conservation than previously appreciated [16]. Notably, frameshift mutation of the longest open reading frame (ORF) in *GAS5* occurs naturally in several inbred mice strains, but has no obvious deleterious phenotypic effects despite predicted marked disruption of polypeptide sequence [17], further arguing against any significant biological role for *GAS5*-encoded protein. Moreover, antibodies raised against *in vitro* translated mouse GAS5 lncRNA fail to detect any protein in cultured cells or in murine tissues [14], perhaps indicating that the polypeptide is expressed at very low levels or actively degraded. In conclusion, based on current evidence, *GAS5* is unlikely to encode a functionally important polypeptide; rather its translation plays an integral role in the regulation of transcript levels, as will be discussed in the next section.

## 4. Regulation of *GAS5* Expression

**Figure 2:**
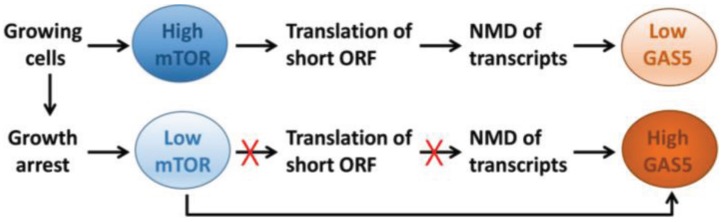
Interplay between mammalian target of rapamycin (mTOR) and nonsense-mediated decay (NMD) regulates cellular GAS5 lncRNA levels.

*Post*-transcriptional mechanisms underlie the increase in *GAS5* expression that accompanies growth arrest due to saturating cell density or nutrient deprivation, whereas transcriptional mechanisms may control *GAS5* expression in differentiating cells [14,18]. The former is perhaps best understood and involves interplay between the mammalian target of rapamycin (mTOR) and nonsense-mediated decay (NMD) pathways [6,19]. Under conditions in which mTOR activity is high, such as in actively growing cells, *GAS5* translation is promoted due to the presence of the 5'-TOP sequence. However due to the short ORF and the presence of multiple termination codons in its sequence, this results in degradation of transcripts via NMD and a lowering of their levels (Figure 2). Conversely, when mTOR activity is low, such as in growth-arrested cells, *GAS5* transcripts are no longer translated and this in turn, prevents NMD (which only occurs for actively translated transcripts), resulting in the accumulation of GAS5 transcripts (Figure 2).

## 5. *GAS5* Expression in Health and Disease

*GAS5* is expressed in the mouse preimplantation embryo from at least the 8-cell stage, with lower abundance at the morula and blastocyst stages [20]. From 8 days post-conception, a spatio-temporal pattern of expression is observed [14]; expression is particularly high in the neural tube in the early embryo and in certain brain structures such as the forebrain and hypothalamus in mid-late gestation. Northern blots of adult tissue revealed relatively high GAS5 levels in brain but low levels in spleen and liver, suggesting an inverse relationship between expression levels and tissue replicative rate [14].

Disturbed *GAS5* expression has been reported in a range of animal pathological models including malformations of neural tube closure [21,22], neuronal hypoxia [23] and the autoimmune disease, systemic lupus erythematosus [24]. More notable however, is the large body of evidence linking *GAS5* dysregulation to a wide array of human cancers (Table 1).

*GAS5* expression is typically reduced in such cancers and clinico-pathological characteristics, such as tumour size, staging, invasion and/or regional lymph node metastasis, show disease-specific inverse correlations with GAS5 levels (Table 1). Cell lines of affected cancers also tend to display reduced *GAS5* expression, especially models of advanced disease (Table 1). Moreover, forced expression of GAS5 lncRNA in such cells inhibits tumour growth in xenograft models (Table 1), mirroring clinical findings. Notably, in an LNCaP xenograft model, GAS5 levels decline concomitant with the acquisition of castrate-resistance, which has important implications for the treatment of advanced prostate cancer. More crucially, for many cancers, low GAS5 lncRNA levels are predictive of poor prognosis (Table 1). Together, these findings support a tumour suppressor role for GAS5 lncRNA *in vivo*.

**Table 1:** GAS5 expression in human cancer. ↓ and ↑ = down- and up-regulated expression, respectively, in the indicated cancer tissues/model systems; → = leading to; TNM = cancer staging system of the International Union for Cancer Control (UICC) which takes into account the primary tumour (T), lymph nodes (N) and metastases (M)—a higher stage indicates a more advanced cancer; FIGO = comparable staging system of the International Federation of Gynecologists and Obstetricians (FIGO) for cancers of the female reproductive organs.

Cancer	Comments	Reference
Breast	↓ Patient tissue/cell lines; poor patient survivalGAS5 inhibits xenograft tumour growth	[11,25,26]
Prostate	↓ Xenografts as cells acquire castrate-resistance	[27]
Head/neck squamous cell	↓ Patient tissue; poor patient survival	[25]
Glioblastoma multiforme	↓ Patient tissue; poor patient survival	[25,28]
Renal clear cell	↓ Patient tissue/cell lines	[29]
Bladder	↓ Patient tissue/cell lines	[30]
Hepatocellular	↓ Patient tissue → increased tumour size/clinical stage/lymph node spread; poor patient survival	[31]
Pancreatic	↓ Patient tissue/cell lines	[32]
Non small cell lung	↓ Patient tissue/cell lines → increased tumour size/TNM stage GAS5 inhibits xenograft tumour growth	[33,34]
Mesothelioma	↑ Patient tissue; ↓ Cell lines	[35]
Gastric	↓ Patient tissue/cell lines → increased tumour size/TNM stage/invasion/regional lymph nodes; poor patient survival GAS5 inhibits xenograft tumour growth	[36,37]
Colorectal	↓ Patient tissue → increased tumour size/TNM stage; lower grade; poor patient survival	[38]
Cervical	↓ Patient tissue → increased FIGO stage/lymph node spread/vascular invasion; poor patient survival	[39]
Adrenocortical	↓ Patient tissue; unrelated to recurrence	[40]
Multiple myeloma	↓ Patient plasma	[41]

Solid tumours apart, GAS5 is also down-regulated in multiple myeloma neoplasms of B-cells but its expression is unaffected in chronic lymphocytic leukemia [41]. In a patient with diffuse large B-cell lymphoma, a chromosomal translocation affecting *GAS5*, t(1;3)(q25;q27), has been described [42]. One breakpoint occurs within the U76 snoRNA sequence and results in a chimeric transcript in which *GAS5* 5'-TOP to exon 3 sequence is spliced to *BCL6* exon 2. Since the fusion transcript contains the ORF of BCL6, then it would not be targeted for NMD, unlike GAS5 lncRNA. Consistent with this finding, *GAS5* was recently identified using a genome-wide strategy as one of several genes that are susceptible to activation-induced cytidine deaminase-mediated rearrangement in murine B-lymphocytes [43]. In addition, chromosomal translocations that produce oncogenic tyrosine kinases may themselves impact upon *GAS5* expression, insofar as GAS5 lncRNA levels are suppressed by BCR-ABL but induced by TEL-JAK2 in murine lymphoid cells [44].

When considering the importance of GAS5 lncRNA in relation to oncogenesis, an important issue to address is the mechanism of the down-regulation that occurs across many cancers, especially since *GAS5* expression inversely correlates with rates of cell proliferation, which themselves tend to be higher in many cancers. In this regard, two recent studies suggest that epigenetic silencing may be responsible in colorectal and NSCLC cell lines, involving hypermethylation of CpG islands in the *GAS5* promoter [33,45]. Moreover, functional studies support a more direct tumour suppressor role for GAS5 lncRNA, as will be discussed in detail in the next section.

## 6. Functional Activities of GAS5 lncRNA

**Figure 3:**
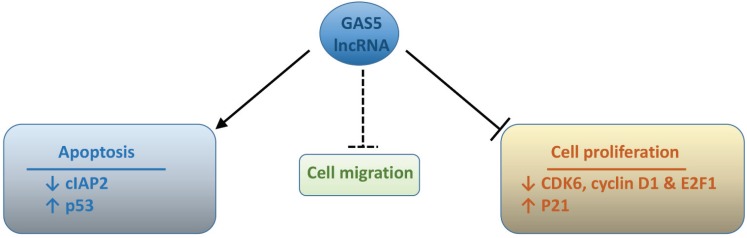
Summary of biological processes that are regulated by GAS5 lncRNA, including possible downstream molecular targets. ↓ and ↑ = down- and up-regulation, respectively.

Clearly GAS5 lncRNA negatively regulates the growth of cell line and xenograft models of various cancers (Table 1). Studies in the latter systems, as well as in untransformed primary cells, demonstrate that GAS5 exerts complementary effects on cell proliferation (inhibitory) and apoptosis (stimulatory), and together these are likely to form the main basis of its tumour suppressor activity *in vivo*. Additional mechanisms centering on cell migration/invasion may also contribute to certain cancers, since GAS5 lncRNA has been recently reported to negatively regulate the migration of renal cell and cervical carcinoma cell lines [29,39], whereas it has no effect on the migration of NSCLC cells [33]. However, the molecular basis of these findings remains to be elucidated. The cellular processes of relevance to cancer that are regulated by GAS5 lncRNA, along with possible downstream molecular targets are summarized in a simplified scheme (Figure 3), which will now be discussed in further detail.

### 6.1. Regulation of Cell Proliferation

As mentioned at the outset, *GAS5* was originally identified in a screen for novel genes involved in growth arrest [5]. Indeed, subsequent work using plasmid constructs and siRNA-mediated silencing approaches in human T-cell lines and normal lymphocytes, has demonstrated that GAS5 lncRNA *per se* is both necessary and sufficient for growth arrest [10]; similar observations have also been reported for a wide range of human epithelial cell lines [29,30,32,33,36,39]. In many cell types, GAS5 lncRNA arrests cells in the G0/G1 phase of the cell cycle; conversely, GAS5 knockdown is associated with a shorter cell cycle length concomitant with a decreased proportion of cells in G0/G1 and an increased proportion of cells in S-phase relative to controls [10,29,30,32,33,35,36,37,38].

Cyclin-dependent kinase 6 (CDK6), which promotes G1 progression and G1/S transition of the cell cycle, is a GAS5-associated protein in bladder cancer cells [30]. *GAS5* silencing increases—and ectopic GAS5 lncRNA expression decreases—CDK6 mRNA and protein levels in cell lines [30,32]; a similar, inverse relationship between GAS5 and CDK6 levels has been reported in bladder and pancreatic cancer tissues [30,32], suggesting that this interaction is operative *in vivo*. Importantly, knockdown of CDK6 expression in these cells blocks the stimulation of cell proliferation consequent upon *GAS5* silencing; conversely, CDK6 overexpression mitigates cell proliferation inhibition by GAS5 lncRNA [30,32]. These effects are partial [30,32], suggestive of additional mechanisms of action of GAS5 in modulating the cell cycle. Indeed, with respect to GAS5 levels, an inverse relationship has been reported both for the cyclin D1 protein, which forms a complex with CDK4/CDK6 and activates these kinases, and for the transcription factor E2F1, which regulates the expression of key cell cycle regulatory genes, whereas a positive relationship exists for the cyclin-dependent kinase inhibitor, p21 [33,36]. In all these cases, regulation is post-transcriptional [33,36], but the underlying molecular mechanism(s) are unknown. Recently, Y-box binding protein 1 (YBX1), a transcriptional activator of p21, has been reported to interact with GAS5 lncRNA in stomach cancer cells [37]. This interaction partly depends on *GAS5* exon 12-derived sequence, results in negative regulation of YBX1 protein turnover, and is accompanied by changes in both mRNA and protein levels of p21 [37]. Like YBX1, the translation of another regulator of cell proliferation, c-Myc (which itself up-regulates cyclins and down-regulates p21), is negatively controlled by GAS5 lncRNA in lymphoma cells [46], as discussed later (see Section 7.3). The relative contribution of these various mechanisms to GAS5-mediated growth arrest remains to be addressed.

### 6.2. Apoptosis Control

The first suggestion that *GAS5* may regulate apoptosis came from functional cloning experiments employing a mouse FDCP1 cDNA library and γ-irradiated mouse W7.2c thymoma cells to isolate novel regulators of cell survival. This resulted in the isolation of a partial *GAS5* sequence which suppressed apoptosis [47]; subsequent experiments in this cell line and in a range of human cell lines/primary cells demonstrated that more complete *GAS5* sequences actually promoted apoptosis [10,11]; mature GAS5 lncRNA is sufficient for this activity [26,29,33,36,48,49,50].

While transfection of GAS5 lncRNA *per se* is sufficient to induce apoptosis in most human cell lines, an exception is the immortalized normal breast cell line, MCF10A [11]. In this cell line, GAS5 has no effect basally but enhances apoptosis consequent upon treatment with cell death-inducing stimuli [11] and, as such, GAS5 can be considered an apoptosis promoter. GAS5 lncRNA also enhances apoptosis induction by external stimuli in those cell lines which are responsive under basal conditions; these effects can be additive [11,48,49] or synergistic [34]. Potent pro-apoptotic activity has been observed for exogenous GAS5 lncRNA constructs in cells which exhibit marked reductions in endogenous *GAS5* expression, including for example triple-negative breast cancer and hormone-resistant prostate cancer cells [48,49], indicating that such cells retain GAS5 sensitivity.

Nevertheless, the influence of reduced *GAS5* expression in reducing cancer cell sensitivity to apoptosis is perhaps of greater clinical relevance, given the widespread down-regulation of *GAS5* expression that occurs across multiple cancer types. Silencing of *GAS5* inhibits the basal apoptotic rate in certain cell lines, but this has negligible impact on culture survival overall, presumably because the basal apoptotic rate tends to be low in most cell lines when grown under favorable conditions [48,49]. More strikingly, *GAS5* silencing attenuates apoptosis induction by a wide range of stimuli, including chemotherapeutic agents with diverse mechanisms of action (Table 2), indicating that GAS5 lncRNA functions in a late and common step of activation of the apoptotic machinery by such agents. Not all treatments show dependence on GAS5 lncRNA for their action, insofar as imatinib-induced death of breast cancer cells is unaffected by *GAS5* silencing [49], however the action of another tyrosine kinase inhibitor, gefitinib, shows GAS5-dependence in lung cancer cells [34]. Crucially, for several agents, quantitative relationships have been noted between GAS5 lncRNA levels and both the extent of apoptosis induction (direct relationship) and the associated loss of culture viability (inverse relationship) [34,48,49], suggesting a key role for GAS5 lncRNA in apoptosis control.

**Table 2:** Apoptotic stimuli which require GAS5 lncRNA for their action.

Treatment	Model	Reference
*DNA damage*		
Ultraviolet-C irradiation	Breast: MCF10A, MCF7, T47D & MDA-MB-231 cells	[11,26,49]
	Prostate: 22Rv1 cells	[48]
	Kidney: HEK293T cells	[11]
*Alkylating agent*		
Cisplatin	Breast: MCF10A, MCF7 cells	[11]
	Kidney: HEK293T cells	[11]
*Taxane*		
Docetaxel	Breast: MCF7 & T47D cells	[49]
	Prostate: 22Rv1 cells	[48]
*Anti-tumour antibiotic*		
Doxorubicin	Breast: MCF10A, MCF7 cells	[11,26]
Mitoxantrone	Prostate: 22Rv1 cells	[48]
*Antimetabolite*		
5-Fluorouracil	Breast: MCF7, T47D	[49]
*Corticosteroid*		
Dexamethasone	Lymphoid: CEM-C7	[10]
*MDM2 antagonist*		
Nutlin-3a	Prostate: 22Rv1 cells	[48]
*EGF-R inhibitor*		
Gefitinib	Lung: A549 cells & xenografts	[34]
*mTOR inhibitor*		
Rapamycin/rapalogues	Lymphoid: CEM-C7, MOLT4, Jeko-1 & Z-138 cells;	[51,52]
	primary T-lymphocytes	
	Prostate: 22Rv1, LNCaP, PC3 & DU145 cells	[53]
AZD8055, BEZ235	Prostate: 22Rv1, LNCaP, PC3 & DU145 cells	[53]

Similar quantitative relationships are characteristic of key apoptosis regulators, such as members of the Bcl-2 family, for which the balance between pro- and anti-apoptotic molecules determines overall sensitivity to apoptotic stimuli [54]. Although the influence of GAS5 lncRNA on Bcl-2 family members has not been studied to-date, several reports demonstrate that other important regulators of sensitivity to apoptosis are targets for this lncRNA. Thus the expression of cIAP2, which is a member of the Inhibitor of Apoptosis Protein (IAP) family and a glucocorticoid–responsive gene, is subject to riborepression by GAS5 lncRNA (see Section 7.1) in some [50,55]—but not all [33]—cells. Furthermore, GAS5 lncRNA enhances the expression of the master regulator of apoptosis, p53, in the NSCLC cell line, A549, *via* an uncharacterised post-transcriptional mechanism [33]. However, GAS5 lncRNA can also induce apoptosis in p53-null NSCLC cells, demonstrating that there is not an absolute requirement for p53 for this activity [33].

## 7. Mechanisms of GAS5 lncRNA Action

To-date three different molecular mechanisms of action have been described for GAS5 lncRNA. In the first two—and best understood—mechanisms, GAS5 lncRNA acts as decoy to either repress steroid receptor-induced transcriptional activation or to inhibit miR-21 action. These decoy activities are quite distinct, involving separate portions of GAS5 lncRNA sequence; to reflect this distinction, the terms “riborepression of steroid hormone receptor action” and “miRNA sponge” are used here. Additional mechanisms, involving direct interaction with CDK6, YBX1 or other proteins identified in interacting partner screens [30,37], for example, are feasible, but require further characterization and are not considered further here.

### 7.1. Riborepression of Steroid Hormone Receptor Action

A major breakthrough in our understanding of GAS5 lncRNA action came with the demonstration that it could directly interact with the DNA binding domain of the glucocorticoid receptor (GR) [50]. This prevents receptor binding to glucocorticoid response elements (GRE) in target genes and consequently blocks the activation of target gene transcription [50]. Through its competition with GREs for binding the GR, GAS5 lncRNA is archetypically considered to act as a decoy molecule or riborepressor of steroid hormone receptor action [50].

Since glucocorticoid receptor-induced transcriptional activation essentially occurs within the nucleus, then riborepression also requires GAS5 lncRNA to be localized in this subcellular compartment; glucocorticoid is thought to enhance the association of cytoplasmic GAS5 lncRNA with the GR, resulting in its co-migration with the ligand-activated GR to the nucleus [50]. Once in the nucleus, it can then compete with DNA GREs located in target genes for the binding of the GR DNA binding domain; compared with DNA GREs, GAS5 lncRNA has *ca*. 2-fold higher binding affinity for the GR DNA binding domain and consequently, stronger displacing activity [50]. A wide range of glucocorticoid-responsive genes which contain GREs in their promoters are subject to such transcriptional repression by GAS5 lncRNA, which notably include negative regulators of apoptosis such as cIAP2 and SGK1 [50]. Furthermore, riborepression appears operative *in vivo* in response to physiological stress, such as serum starvation, which elevates GAS5 lncRNA levels [50].

The sequence of GAS5 lncRNA that interacts with the GR DNA binding domain resides within exon 12-encoded sequence and comprises a hairpin structure that contains two GRE-like sequences, termed GRE-1 and GRE-2, which are complementary to each other [50]. Notably, GAS5 lncRNA can also interact with other members of the steroid nuclear receptor superfamily members, particularly 3-keto steroid receptors, such as the mineralocorticoid receptor, the androgen receptor and progesterone receptor-A, which can bind DNA-activating GREs, but not estrogen receptor α or peroxisome proliferator-activated receptor δ [16,50]. Thus, GAS5 lncRNA may riborepress the action of certain other steroid hormones in addition to glucocorticoids.

A recent study has provided more detailed structural information about GAS5 lncRNA/glucocorticoid receptor interaction, including the identity of both the bases within the stem-loop HRE mimic (HREM) sequence of GAS5 lncRNA and the amino acids within the DNA binding domain of GR that play crucial roles [16]. Interestingly, this interaction occurs in a similar manner to which the GR interacts with activating DNA GREs [16]. Notably mutation (G549A) of one of the two GC pairs in the GAS5 lncRNA HREM sequence that are critical for contacting amino acid residues in helix 1 of the GR DNA binding domain is sufficient to inhibit the GR/GAS5 lncRNA interaction, GAS5 lncRNA-mediated riborepression and the functional effects of GAS5 lncRNA on several different cell types, albeit in a cell type-dependent manner [16]. Thus, compared with the wild-type sequence, mutant (G549A) GAS5 lncRNA is unable to riborepress androgen-mediated induction of several androgen target genes in prostate cancer cells, and its ability to induce apoptosis is either abolished (in prostate and breast cancer cell lines) or attenuated (lymphoid cell lines) [16]. The latter observations indicate that riborepression may constitute the major molecular mechanism of GAS5 lncRNA action in promoting apoptosis in epithelial cell types, but additional mechanisms may contribute to this phenomenon in lymphoid cells.

### 7.2. miRNA Sponge

GAS5 lncRNA has recently been identified as a novel target for miR-21, which functions as an oncogene in various types of solid tumour and lymphoma [26]. GAS5 lncRNA is negatively regulated by miR-21, as are several other tumour suppressor genes, including PTEN, maspin and PDCD4 [26]. This regulation forms one arm of a reciprocal feedback loop, since GAS5 lncRNA itself negatively regulates miR-21 [26]. Exon 4-derived sequence of *GAS5* is thought to contain a miR-21 binding site [26], so that GAS5 lncRNA may function as a miRNA sponge; this sequence is also important for the reciprocal negative regulation of GAS5 lncRNA by miR-21 [26]. GAS5 lncRNA is without effect on either pri- or pre-miR-21, while miR-21 has no effect on *GAS5*-encoded snoRNAs, indicating that post-transcriptional mechanisms underpin this regulatory loop [26], albeit further work is required for more detailed understanding. Finally, GAS5 lncRNA and miR-21 levels are negatively correlated in xenografts of MCF7 cells overexpressing GAS5 lncRNA, as well as in clinical breast cancer specimens, suggesting that this mechanism is of clinical importance [26], albeit the cause and effect relationship of the latter observations is unclear.

### 7.3. Regulation of Translation

GAS5 lncRNA has recently been reported to co-precipitate with the eukaryotic translation initiation complex in lymphoma cells, suggestive of a possible role in gene translation [46]. Two novel RNA binding motifs have been identified in eukaryotic translation initiation factor 4E (eIF4E) which mediate this interaction, but the identity of the GAS5 lncRNA interacting sequence is unknown [46]. GAS5 lncRNA negatively and specifically regulates the translation of the eIF4E downstream target gene, c-Myc, without affecting either other target genes (such as Mcl1, survivin or Bcl2) or global protein translation. Direct interaction of GAS5 lncRNA with c-Myc RNA is thought to be involved in this regulation, possibly inhibiting the entrance of c-Myc into the polysome [46].

## 8. Conclusions and Perspectives—Novel Clinical Translational Opportunities?

It is clear from the foregoing that *GAS5* expression is reduced in multiple tumours and can impact patient outcomes. The role of GAS5 lncRNA in promoting both growth arrest and apoptosis is likely to constitute the basis of its tumour suppressor gene function. Moreover, decreased levels of GAS5 lncRNA are likely to have major implications for the treatment of such cancers, since radiation therapy and many chemotherapies usually depend upon the efficient engagement of the apoptotic machinery for their action [56,57]. This argument is underscored by the quantitative relationships that exist between GAS5 lncRNA levels and both patient prognosis and apoptosis induction in preclinical models (see Section 5 and Section 6.2). Indeed, the latter observations indicate that even small reductions in endogenous *GAS5* expression may adversely impact the responses of some cancer cell types to certain death-inducing stimuli, including conventional chemotherapeutic agents. Cell lines representative of advanced cancers (hormone-independent breast and prostate cancer, for example) often exhibit low levels of GAS5 expression (relative to untransformed cells or models of early stage disease) [26,48,49] but crucially, these retain the ability to respond to ectopic GAS5 lncRNA [48,49]. It therefore follows that the restoration of GAS5 lncRNA levels is likely to improve patient outcomes.

One simple way to achieve this may be to exploit the natural physiological mechanisms that control cellular GAS5 levels, through the use of mTOR inhibition (see Section 4), especially since mTOR inhibitors, either alone or in combination with other agents, are already in use in the clinic or the subject of multiple clinical trials for a range of cancers [58,59]. However, drug resistance can be an issue with these agents [58,59], which led us to address the feasibility of their use for modulating GAS5 lncRNA levels in a range of preclinical models of breast and prostate cancer, including hormone insensitive disease [49,53]. Thus, rapamycin and the rapalogues, everolimus and temsirolimus, were able to both enhance GAS5 lncRNA levels and inhibit culture growth in hormone-sensitive cell lines, whereas hormone-independent cells showed resistance to these agents as well as to newer generation inhibitors, such as combined mTORC1/mTORC2 and dual PI3K/mTOR inhibitors [49,53]. The hormone-insensitive cell lines express lower levels of GAS5 lncRNA than the hormone-sensitive cells [49,53] and further functional studies have demonstrated that GAS5 lncRNA is itself required for mTOR inhibitor action in prostate cancer cells, as is the case in lymphoid cells (Table 2). Thus, while mTOR inhibition may serve as a useful strategy to enhance GAS5 lncRNA levels to promote the death of hormone-sensitive breast and prostate cells, it is unlikely to be successful in hormone-insensitive disease for which there is a more pressing need to develop novel targeted therapies.

Better understanding of the mechanisms that underlie GAS5 silencing in advanced cancer may assist in this regard. As discussed earlier (Section 5), emerging evidence suggests that epigenetic mechanisms may be important, and specific targeting of these may offer novel, mTOR inhibitor-independent approaches to enhance GAS5 lncRNA levels. An alternative approach may be to use chemotherapeutic agents which are independent of GAS5 lncRNA for their action; for example, GAS5 silencing has no effect on imatinib-induced cell death in breast cancer cells [49]. While imatinib *per se* may not be suitable for the treatment of breast cancer [60], it is possible that other agents exist which are GAS5-independent, and further drug screening is required to identify these.

Therapeutics aside, there is potential for GAS5 lncRNA to serve as a biomarker both diagnostically and for monitoring therapeutic responses, given the correlations between GAS5 levels and clinico-pathological characteristics, that have been reported for several cancers (Table 1). Such monitoring may require sampling of body fluids only for the isolation of exosomes, since a preclinical study has shown that GAS5 lncRNA is enriched in these cell-derived vesicles, as are several other lncRNA molecules [61]. Although the analysis of lncRNA in urinary exosomes has recently revealed that GAS5 lncRNA is not a suitable biomarker for distinguishing prostate cancer from benign prostatic hyperplasia (unlike lincRNA-p21) [62], further work is required in a wider range of cancers, particularly those in which clear correlations exist between cellular GAS5 lncRNA levels and indices of disease progression (Table 1). Finally, given the relationships between mTOR inhibition and GAS5 lncRNA levels discussed above in prostate, breast and lymphoid cells, GAS5 lncRNA may potentially serve as a predictive biomarker for therapies targeting mTOR, to facilitate the selection of patients who are likely to achieve maximum benefit from such treatments, given the associated problems of drug resistance.
